# Effects of a fermented cotton straw-apple pomace mixture on growth performance, rumen microbial community, and metabolome in beef cattle

**DOI:** 10.3389/fmicb.2025.1747833

**Published:** 2026-01-02

**Authors:** Qikai Liu, Ruohui Li, Bao Wang, Shihong Mi, Chengcheng Wang, Ning Zhang, Xiaoping Chen, Shuaibin Zhou, Tengyu Wang, Xinyi Wang, Xinwen Sun, Dengke Hua, Xinfeng Wang

**Affiliations:** 1 Department of Animal Science and Technology, Shihezi University, Xinjiang, China; 2 Xinjiang Production and Construction Corps 4th Division Chuangjin Agricultural Development Group Co., Ltd., Xinjiang, China; 3 Xinjiang Tianyu Biotechnology Co., Ltd., Tumushuke, China

**Keywords:** apple pomace, cotton straw, enzyme activity, metabolome, microbial fermentation, rumen microbiota

## Abstract

**Introduction:**

The utilization of agricultural by-products as feed plays a significant role in reducing resource waste and promoting sustainable development of the livestock industry. This study investigated the effects of replacing corn silage with a fermented cotton straw-apple pomace mixture in beef cattle diets.

**Methods:**

Twenty beef cattle were randomly assigned to two groups: a control (CON) group fed a basal diet and a treatment (TRE) group fed a diet in which corn silage was replaced by the fermented mixture. We assessed growth performance, rumen fermentation parameters, fibrolytic enzyme activities, bacteria community structure, and metabolite profiles.

**Results:**

Compared to the CON group, the TRE group showed reductions in average daily gain (ADG) and dry matter intake (DMI) by 25.42 and 18.79%, respectively (*p* < 0.05). The concentrations of ruminal volatile fatty acids (VFAs) were also significantly lower (ranging from 9.63 to 17.01% reduction; *p* < 0.05). The activities of cellulase, hemicellulase, and cellobiohydrolase were significantly decreased in the TRE group (by 13.22, 38.80, and 13.66%, respectively; *p* < 0.05). The fermented mixture also altered the rumen microbial composition: the relative abundances of Anaeroplasma and Pyramidobacter were higher in the TRE group, whereas those of Anseongella, Holdemania, and Acetoanaerobium were lower. Non-targeted metabolomics analysis revealed significant changes in the rumen metabolite profile of cattle fed the fermented mixture; notably, ferulic acid concentrations were significantly higher in the TRE group than in the CON group. Spearman correlation analysis indicated that Anseongella was significantly positively correlated with ADG, while Pyramidobacter was significantly negatively correlated with ADG and with the activities of cellulase, hemicellulase, cellobiohydrolase. Anaeroplasma was negatively correlated with those enzyme activities and was also significantly associated with ferulic acid and many other differential metabolites.

**Discussion:**

In conclusion, replacing corn silage with the fermented cotton straw-apple pomace mixture reduced ADG, VFA concentrations, and ruminal fibrolytic enzyme activities in beef cattle; these effects may be related to changes in specific rumen bacteria and metabolites.

## 1 Introduction

Cotton is widely cultivated globally because of its major role in the textile industry. Global cotton planting area was 34.5 million hectares in 2021 (Cai et al., 2024). China was the world’s largest cotton producer from 1983 to 2015 (Feng et al., 2022). Large quantities of cotton by-products are generated during harvesting; cotton straw alone amount to approximately 40 million tons annually (Mou et al., 2023). Cotton straw contains more crude protein content compared to cereal crop straw, making it a potential feed resource (Rehemujiang et al., 2019). However, cotton straw is high in cellulose, hemicellulose, and lignin (Xiong et al., 2014), which lowers palatability and digestibility. It also contain free gossypol, which can accumulate in animals after prolonged ingestion and cause liver and kidney damage (Rehemujiang et al., 2019). Apples are among the world’s most popular fruits. According to the Food and Agriculture Organization of the United Nations (FAO) statistics, global apple production reached 95 million tons in 2022 (Bedriñana et al., 2024). A portion is processed into apple juice, producing about 25% apple pomace as by-product (Shalini and Gupta, 2010). Apple pomace is rich in polyphenols, vitamins, and other nutrients and is widely used in animal feed (Zhang et al., 2021); many studies have reported health benefits for animals (Guil-Guerrero et al., 2016; Ravn-Haren et al., 2018). However, its high moisture content makes it prone to spoilage and difficult to store and transport (Dhillon et al., 2013), which limits its application as feed.

Microbial fermentation is a widely recognized approach for converting agricultural by-products into valuable feed products (Wu et al., 2025). In this process, microorganisms degrade components such as starch and cellulose in the substrate, producing metabolites including lactic acid and acetic acid (Garrido-Galand et al., 2021), and generating enzymes including cellulases (Zhu et al., 2024), thereby enhancing the nutritional value of raw materials and degrading anti-nutritional factors (Cui et al., 2023; Ibarururi et al., 2021). Previous studies have demonstrated that microorganisms can reduce free gossypol in cottonseed meal and significantly degrade cellulose, hemicellulose, and lignin in crop straw (Wang et al., 2021; Zhang et al., 2022; Zhao et al., 2020; Chen et al., 2024). Therefore, microbial fermentation can be applied to lower their cellulose, hemicellulose, lignin, and free gossypol contents in cotton straw. However, cotton straw is low in soluble carbohydrates and moisture, which may impair fermentation. Apple pomace, by contrast, is rich in soluble carbohydrates and moisture, co-fermentation of cotton straw with apple pomace could therefore improve fermentation outcomes.

Previous studies have shown that producing fermented feed from agricultural by-products for animal feeding does not adversely affect production performance and can reduce feeding costs (Suriyapha et al., 2022; Gunun et al., 2023). A fermented cotton straw-apple pomace mixture would promote efficient use of these by-products and minimize resource waste, while lowering livestock production costs. However, its specific effects on animal growth and development remain unclear.

The rumen microbiota composed of bacteria, archaea, fungi, and protozoa is essential to ruminant digestion (Cristobal-Carballo et al., 2021). It degrades cellulose and hemicellulose and produces volatile fatty acids (VFAs) that provide energy to the host (Zhou et al., 2018). The rumen microbiota also utilize dietary proteins and non-protein nitrogen to synthesize microbial protein (MCP) for host use. Consequently, rumen microbiota are crucial for ruminant growth and development. Dietary composition significantly influences the structure and functions of the rumen microbiota (Zhang et al., 2025; Cui et al., 2022). However, it remains unclear what impact replacing corn silage with a fermented cotton straw-apple pomace mixture would have on the composition and function of rumen microbiota.

This experiment aims to evaluate the effects of substituting corn silage in a total mixed ration with a fermented cotton straw-apple pomace mixture on growth performance, rumen fermentation profile, ruminal enzyme activity, microbial community, and metabolite profile. We hypothesize that feeding beef cattle a fermented cotton straw-apple pomace mixture would alter rumen microbial composition to some degree but would not disturb microbial homeostasis or adversely affect growth performance.

## 2 Materials and methods

### 2.1 Animals, diets and experimental design

This study was conducted at Chuangjin Animal Husbandry Co., Ltd., in Kekedala City, Xinjiang Uygur Autonomous Region. All animals and procedures in this study were approved by Biology Ethics Committee of Shihezi University (approval number A2025-987).

Twenty healthy Xinjiang Brown beef cattle of similar body weight (341.76 ± 16.32 kg) and age were randomly allocated into two treatment groups (10 animals per group) with each group housed in a separate pen. The basal diet was formulated according to the “National Beef Cattle Feeding Standard (NY/T 815–2004).” The basal diet composition and nutrient profile are presented in Table 1. The control (CON) group received the basal diet, while the treatment (TRE) group received a diet in which corn silage was replaced with a fermented cotton straw-apple pomace mixture. Diets were isonitrogenous and isoenergetic. The fermented feed mixture was prepared by mixing cotton straw and apple pomace at a ratio of 7:3 (on dry matter basis), supplemented with 0.1% urea, 0.2% salt, and 0.5% composite microbial inoculant (containing *Lactobacillus plantarum* ≥ 1.5 × 10^6^ CFU/mL, *Candida utilis* ≥ 3.0 × 10^5^ CFU/mL, *Saccharomyces cerevisiae* ≥ 1.0 × 10^6^ CFU/mL, *Candida tropicalis* ≥ 1.2 × 10^5^ CFU/mL, *Bacillus subtilis* ≥ 2.0 × 10^6^ CFU/mL, and *Geotrichum candidum* ≥ 1.0 × 10^4^ CFU/mL) (Zhou et al., 2025). Water was added to adjust the moisture content to approximately 65%. Finally, the mixture was fermented anaerobically in a silo for 60 days. The feeding trial lasted 70 days, consisting of a 10-day adaptation period followed by a 60-day experimental period. Each bull was weighed on the first and last day of the trial to calculate the average daily gain (ADG). Animals were fed twice daily at 10:00 a.m. and 5:00 p.m., with ad libitum access to feed and water. Daily feed offering was recorded, and residual feed was collected for three consecutive days at the beginning, middle, and end of the trial to determine dry matter intake (DMI).

**Table 1:** Ingredients and nutritional composition of the diets.^a^

Items	CON	TRE
Ingredient (%, DM basis)		
Corn	28	30
Cottonseed meal	5	2
Rapeseed meal	7	12
Corn silage	23	0
Fermented mixture of cotton straw and apple pomace	0	23
Corn straw	8	8
Wheat straw	11	7
Wrapped baled alfalfa	15	15
Salt	0.5	0.5
Sodium bicarbonate	1	1
Calcium bicarbonate	0.5	0.5
Premix^b^	1	1
Nutrition levels^c^		
Metabolic energy, MJ/Kg	10.78	9.83
Crude protein, %	13.31	13.48
Neutral detergent fiber, %	34.61	34.15
Acid detergent fiber, %	15.31	17.04
Calcium, %	0.89	0.83
Phosphorus, %	0.35	0.38

a CON refers to the control group received corn silage; TRE denotes the treatment group received a fermented mixture of cotton straw and apple pomace.

b Premix per kg diet contains: vitamin A, 100 KIU; vitamin D3, 30 KIU; vitamin E, 600 IU; Fe, 2000 mg; Cu, 200 mg; Zn, 1,000 mg; Mn, 500 mg; Se, 10 mg; Co, 20 mg; I₂, 20 mg.

c ME was calculated, others were measured.

### 2.2 Sample collection

On the final day of the experiment, rumen fluid was collected from each beef cattle 2 h before the morning feeding using a sterile gastric tube, with the initial 50 mL discarded. The collected fluid was immediately filtered through four layers of gauze and pH was measured (HI-9024C, HANNA Instruments, United States). Filtered rumen fluid was aliquoted into three 10 mL cryovials and one 50 mL centrifuge tube. The cryovials were stored at −80 °C, and the centrifuge tube was maintained at −20 °C for subsequent parameter analysis.

### 2.3 Rumen fermentation profile and enzyme activity

Rumen fluid stored at −20 °C was thawed for the analysis of fermentation profile, including ammonia nitrogen (NH₃-N), microbial crude protein (MCP), and volatile fatty acid (VFA) concentrations. NH₃-N concentration was determined by the colorimetric method (Chaney and Marbach, 1962), MCP content was determined using a commercial kit (Beijing Solarbio Science and Technology Co., Ltd., Beijing, China), and VFA concentrations were determined by high-performance gas chromatography (Agilent-7890A, United States).

One 10 mL cryovial was thawed for enzyme activity assays. The activities of cellulase, cellobiohydrolase, and xylanase were determined immediately using commercial kits (Suzhou Geruisi Biotechnology Co., Ltd., Suzhou, China).

### 2.4 16S rRNA gene sequencing and data analysis

Total genomic DNA was extracted from rumen fluid samples using the E. Z. N. A.® Stool DNA Kit (Omega Bio-tek, Norcross, GA, United States) following the manufacturer’s instructions. The V4–V5 region of the bacterial 16S rRNA gene was amplified by PCR under the following conditions: initial denaturation at 95 °C for 2 min; 25 cycles at 95 °C for 30 s; annealing at 55 °C for 30 s; extension at 72 °C for 30 s; a final extension at 72 °C for 5 min. The primers used were 515F (5′-barcode-GTGCCAGCMGCCGCGG-3′) and 907R (5′-CCGTCAATTCMTTTRAGTTT-3′), where the barcode represents an eight-base sample-specific sequence. Each PCR was performed in triplicate 20 μL reactions containing 4 μL of 5 × FastPfu Buffer, 2 μL of 2.5 mM dNTPs, 0.8 μL of each primer (5 μM), 0.4 μL of FastPfu Polymerase, and 10 ng of template DNA. Amplicons were separated on 2% agarose gels and purified using the AxyPrep DNA Gel Extraction Kit (Axygen Biosciences, Union City, CA, United States). Purified amplicons were quantified with Qubit® 3.0 (Life Invitrogen, United States), and every 24 amplicons with distinct barcodes were pooled in equimolar ratios. The pooled DNA was used to construct an Illumina paired-end library. Then the amplicon library was paired-end sequenced (2 × 300) on an Illumina platform (Shanghai BIOZERON Biotech. Co., Ltd). Raw sequencing data have been deposited in the NCBI under accession number PRJNA1332918.

Raw FASTQ files were processed using Trimmomatic (version 0.39; Bolger et al., 2014) and custom Perl scripts for demultiplexing. Processing criteria included: (i) trimming reads when the average quality score fell below 20 within a 10 bp sliding window and discarding reads shorter than 50 bp after trimming; (ii) removing reads with inexact barcode matches, more than 2 nucleotide mismatches in the primer region, or ambiguous bases; (iii) assembling overlapping reads with a minimum overlap of 10 bp. Passed sequences were dereplicated and processed with the DADA2 algorithm to identify sequence variants (Callahan et al., 2016). Trimming and filtering used a maximum of two expected errors per read (maxEE = 2). After merging and chimera filtering, taxonomic assignment was performed using the uclust algorithm (Edgar, 2010) against the Silva (SSU138.1) (Quast et al., 2013) 16S rRNA database^1^ with an 80% confidence threshold.

Rarefaction curves were generated using Mothur v1.21.1 (Schloss et al., 2009) to verify sequencing depth adequacy. Subsequent analysis was performed in R (version 4.4.2). Alpha diversity was assessed with the R package vegan (Dixon, 2003) using the Shannon, Simpson, and Chao1 indices. Beta diversity was examined by principal coordinate analysis (PCoA) based on Bray–Curtis distances and tested by permutational multivariate analysis of variance (PERMANOVA). Differentially abundant taxa were identified by linear discriminant analysis (LDA) effect size (LEfSe) with LDA score ≥ 2.0 and *p* < 0.05 using the R package microeco (Liu et al., 2021).

### 2.5 LC/MS-based metabolomics data analysis

Rumen fluid samples were thawed on ice, and 1.0 mL of each sample was lyophilized. Lyophilized material was extracted with 300 μL of 80% methanol, vortexed, sonicated for 30 min at 4 °C, incubated at −40 °C for 1 h, vortexed again for 30 s, and kept at 4 °C for 0.5 h. After centrifugation at 12,000 rpm and 4 °C for 15 min, the supernatant was transferred into a new tube and stored at −40 °C for 1 h, followed by a second centrifugation under the same conditions. Then, 200 μL of the supernatant was mixed with 5 μL internal standard (0.20 mg/mL dichlorophenylalanine) and transferred to an injection vial.

Chromatographic separation was achieved using water with 0.05% formic acid and acetonitrile as mobile phase. Column temperature was maintained at 40 °C, flow rate 0.300 mL/min, and injection volume 5 μL. Gradient elution (water:acetonitrile) was set as follows: 0–1 min, 95:5; 12.5–13.5 min, 5:95; 13.6–16 min, 95:5. Mass spectrometry used electrospray ionization (ESI) with voltages of 3.0 kV (positive mode) and 3.2 kV (negative mode). Heater temperature was 300 °C; sheath gas, auxiliary gas, and sweep gas flows were 45, 15, and 1 arb, respectively. Capillary temperature was 350 °C.

Raw MS files were processed in Compound Discoverer 3.1 for spectral processing and database matching to identify and quantify metabolites. Data preprocessing included: (1) retaining peak area data with no more than 50% missing values in any single group or across all groups, (2) imputing missing values using the minimum value divided by two method, and (3) normalizing the data based on the total ion current (TIC) of each sample. Orthogonal partial least squares-discriminant analysis (OPLS-DA) was performed using the R package ropls (Thévenot et al., 2015), and validated by 200 permutation tests. Differential metabolites were identified based on *t*-tests (*p* < 0.05) and variable importance in projection (VIP) > 1. Differential metabolites were visualized by cluster heatmaps, and pathway enrichment analysis was performed using MetaboAnalystR (Pang et al., 2024).

### 2.6 Statistical analysis

An independent-sample *t*-test was performed using SPSS 27.0 software to compare the data on growth performance, rumen fermentation profile, and rumen enzyme activities between the CON and TRE groups; *p* < 0.05 was considered statistically significant. Procrustes analysis was used to assess the similarity between the microbiome and metabolome profiles. Spearman correlations among differentially abundant bacteria, differential metabolites, rumen enzyme activities, and ADG were carried out using the R (version 4.4.2). Correlation networks were generated using the R (version 4.4.2), and heatmaps plotted with complexHeatmap (Gu et al., 2016).

## 3 Results

### 3.1 Growth performance

The ADG, DMI, and feed conversion ratio (FCR) are presented in Table 2. ADG and DMI in the TRE group were significantly lower than those in the CON group (*p* < 0.05). FCR did not differ significantly between groups (*p* > 0.05).

**Table 2:** Effects of different diets on the growth performance of beef cattle.

Items	CON	TRE	SEM	*P*-value
ADG (kg)	0.59	0.44	0.03	0.003
DMI (kg/d)	8.78	7.13	0.31	0.004
FCR	15.32	16.21	0.69	0.531

ADG, average daily gain; DMI, dry matter intake; FCR, feed intake to body weight gain ratio. CON refers to the control group received corn silage; TRE denotes the treatment group received a fermented mixture of cotton straw and apple pomace.

### 3.2 Rumen fermentation characteristics

As shown in Table 3, the ruminal concentrations of MCP, NH_3_-N, acetate, propionate, isobutyrate, butyrate, and total VFA content in the TRE group were significantly lower than those in the CON group (*p* < 0.05). No statistically significant differences were observed in valerate or isovalerate concentrations, the acetate to propionate ratio, or ruminal fluid pH (*p* > 0.05).

**Table 3:** Effects of different diets on rumen fermentation profile of beef cattle.

Items	CON	TRE	SEM	*P*-value
pH	6.71	6.84	0.06	0.265
NH_3_-N (mg/dL)	6.69	5.85	0.19	0.021
MCP (mg/mL)	19.52	14.10	0.87	<0.001
Total VFA (mmol/L)	69.30	62.80	1.02	<0.001
Acetate (mmol/L)	45.80	41.93	0.89	0.024
Propionate (mmol/L)	13.53	11.91	0.36	0.019
Isobutyrate (mmol/L)	0.41	0.34	0.02	0.042
Butyrate (mmol/L)	7.03	6.23	0.18	0.023
Isovalerate (mmol/L)	1.36	1.24	0.03	0.066
Valerate (mmol/L)	1.24	1.19	0.03	0.353
Acetate/propionate	3.44	3.57	0.12	0.629

MCP, microbial crude protein; VFA, volatile fatty acid. CON refers to the control group received corn silage; TRE denotes the treatment group received a fermented mixture of cotton straw and apple pomace.

### 3.3 The effects on rumen enzyme activity

The effects of different treatments on ruminal enzyme activities in beef cattle are presented in Table 4. Activities of cellulase, cellobiohydrolase, and xylanase in the TRE group were significantly lower than those in the CON group (*p* < 0.05).

**Table 4:** Effects of different diets on rumen enzyme activity of beef cattle.

Items	CON	TRE	SEM	*P*-value
Cellulase (U/mL)	1004.95	872.12	24.69	0.004
Cellobiohydrolase (U/mL)	504.55	308.79	31.68	0.001
Xylanase (U/mL)	113.25	97.78	2.87	0.004

CON refers to the control group received corn silage; TRE denotes the treatment group received a fermented mixture of cotton straw and apple pomace.

### 3.4 Rumen bacterial communities

**Figure 1:**
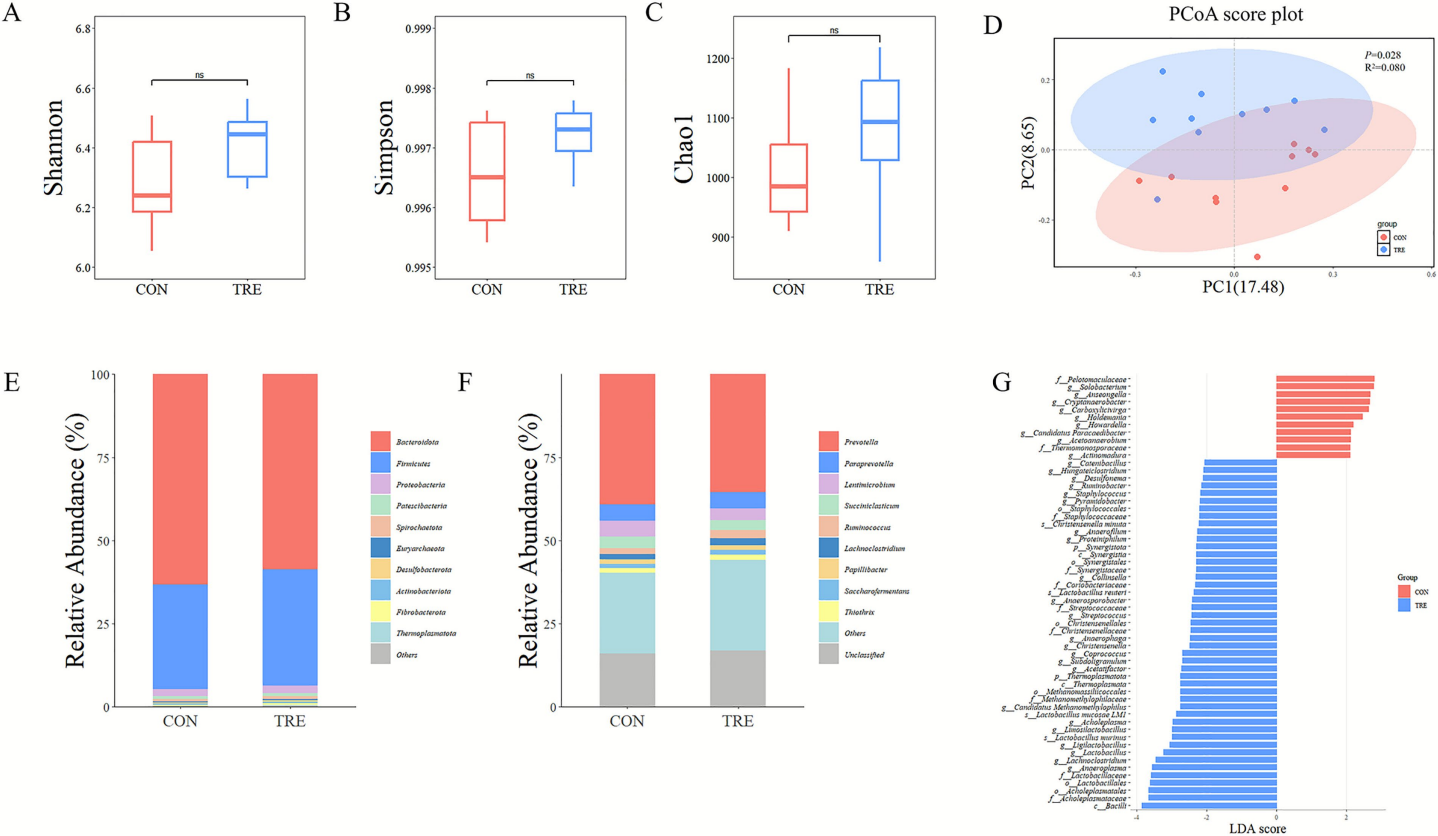
Effect of different diets on rumen bacterial community in beef cattle. **(A–C)** Alpha diversity analysis using the Shannon, Simpson, and ACE indices. **(D)** Principal coordinate analysis (PCoA) based on Bray-Curtis distance. **(E,F)** Bacterial composition at the level of phylum and genus. **(G)** Linear discriminant analysis effect size (LEfSe) analyses (LDA > 2, *p* < 0.05). CON refers to the group fed corn silage, TRE refers the group fed a fermented cotton straw-apple pomace mixture.

High-throughput 16S rRNA sequencing yielded 9,509 amplicon sequence variants (ASVs). Alpha diversity indices (Chao1, Shannon, Simpson) did not differ significantly between CON and TRE groups (*p* > 0.05) (Figures 1A–C). Beta diversity analysis by PCoA showed clear separation between the two groups (Figure 1D), which was further confirmed by PERMANOVA (*p* < 0.05). At the phylum level, Bacteroidota and Firmicutes were the dominant phyla in both groups (Figure 1E). At the genus level, *Prevotella* was the most abundant genus in both groups (Figure 1F).

LEfSe identified the differentially abundant bacteria between the two groups (Figure 1G). The taxa Bacilli, Acholeplasmatales, and Lactobacillales were significantly more abundant in the TRE group (*p* < 0.05), whereas Thermomonosporaceae, *Acetoanaerobium*, and *Pelotomaculaceae* were significantly less abundant (*p* < 0.05). At the genus level, 32 genera differed significantly: nine genera, including *Solobacterium*, *Anseongella*, and *Holdemania*, were lower in the TRE group (*p* < 0.05); 23 genera, such as *Anaeroplasma*, *Lachnoclostridium*, and *Lactobacillus*, were higher in the TRE group (*p* < 0.05).

### 3.5 Rumen metabolomics

**Figure 2:**
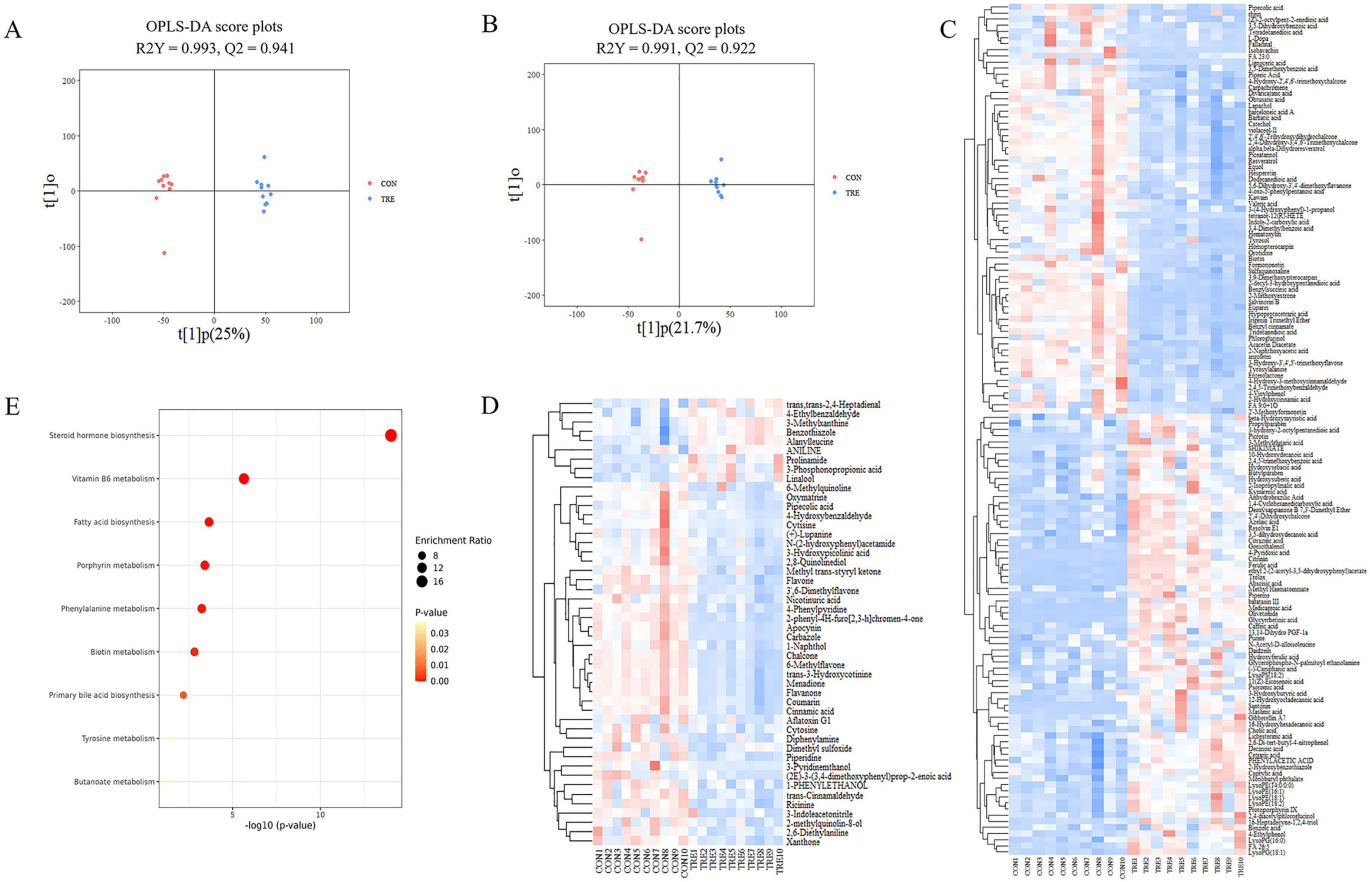
Effect of different diets on rumen metabolism in beef cattle. **(A,B)** OPLS-DA score plots in negative and positive ion modes. **(C,D)** Clustering heatmap of differential metabolites in negative and positive ion modes (VIP > 1, *p* < 0.05). **(E)** KEGG pathway analysis. The *x*-axis represents pathway impact, and the y-axis represents pathway enrichment. The size of the circles corresponds to the degree of pathway enrichment, with larger circles indicating greater enrichment. The color intensity reflects the pathway impact values, with darker colors indicating higher impact. Colors closer to red represent smaller *p*-values. CON refers to the group fed corn silage, TRE refers to the group fed a fermented cotton straw-apple pomace mixture.

The OPLS-DA showed clear separation between CON and TRE metabolic profiles (Figures 2A,B). Model evaluation by 7-fold cross-validation yielded R^2^Y = 0.991 and Q^2^ = 0.922 in positive ion mode and R^2^Y = 0.993 and Q^2^ = 0.941 in negative ion mode. Permutation testing (200 iterations) produced *p*-values < 0.01, indicating no overfitting and significant group separation. Based on VIP > 1 and *p* < 0.05, a total of 187 differential metabolites were identified, with 139 in negative ion mode and 48 in positive ion mode (Figures 2C,D). KEGG enrichments analysis demonstrated that upregulated metabolites in the TRE group were primarily involved in vitamin B6 metabolism, fatty acid biosynthesis, and porphyrin metabolism pathways, while downregulated metabolites were associated with steroid hormone biosynthesis and tyrosine metabolism (Figure 2E).

### 3.6 Correlation analysis between ADG, rumen enzyme activity, differential bacterial genera, and differential metabolites

**Figure 3:**
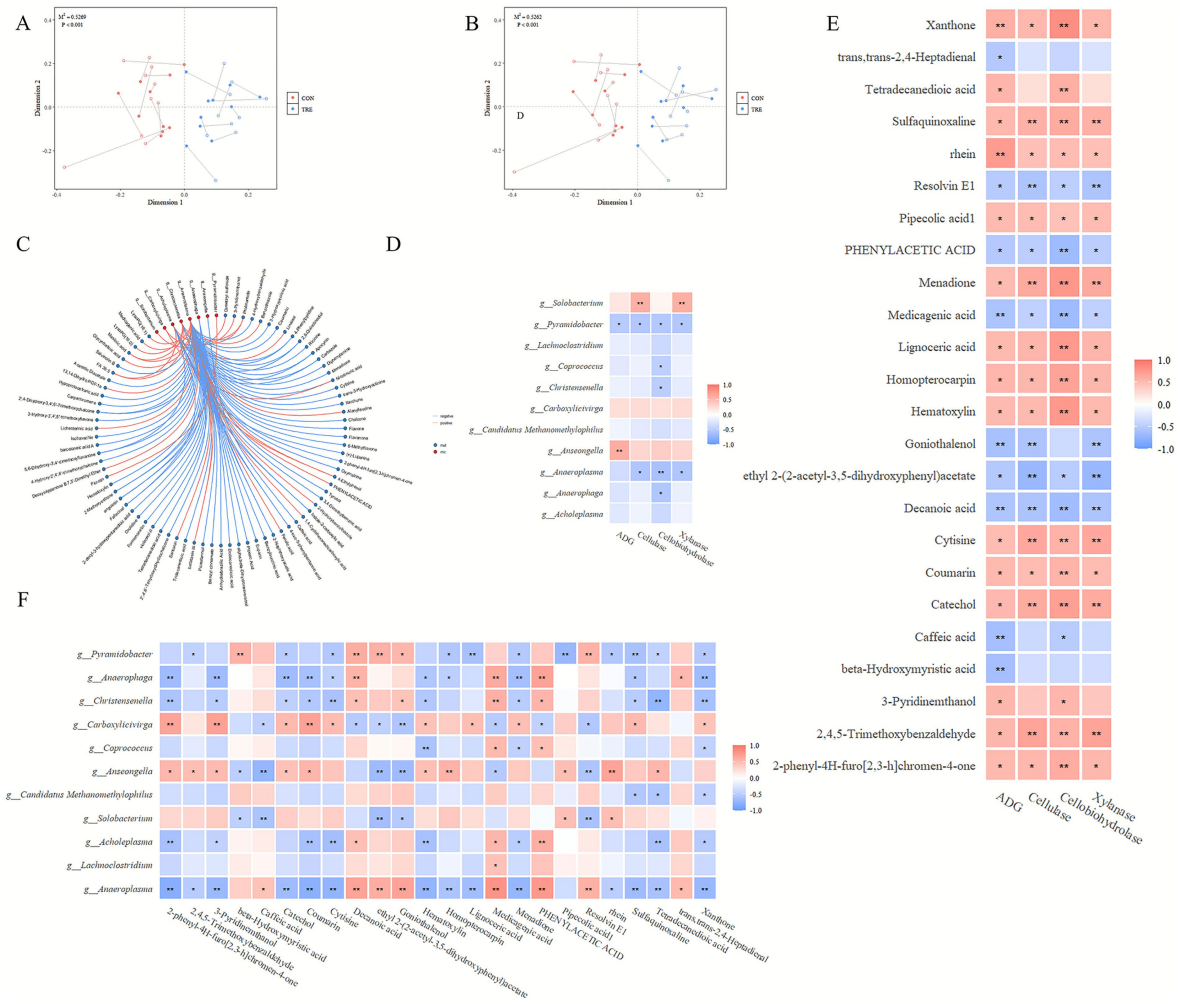
Correlation analysis. **(A,B)** Procrustes analysis of metabolites and microorganisms in negative and positive ion modes. **(C)** Spearman correlation analysis between differentially metabolites and differentially abundant bacterial genera. Blue nodes represent metabolites, red nodes represent bacteria, blue edges indicate significant negative correlations (*r* ≤ −0.7, *p* < 0.05), and red edges indicate significant positive correlations (*r* ≥ 0.7, *p* < 0.05). **(D)** Spearman correlation heatmap of average daily gain, cellulase, cellobiohydrolase, xylanase, and differentially abundant bacterial genera. **(E)** Spearman correlation heatmap of average daily gain, cellulase, cellobiohydrolase, xylanase, and differentially abundant metabolites. **(F)** Spearman correlation heatmap between differentially abundant bacterial genera and metabolites. CON refers to the group fed corn silage, TRE refers the group fed a fermented cotton straw-apple pomace mixture. *p < 0.05, **p < 0.01.

Procrustes analysis between metabolomic (positive and negative modes) and microbial data revealed a significant overall association (Figures 3A,B). Spearman correlation analysis between differential bacterial genera and differential metabolites identified 97 significant correlations (*p* < 0.05, |*r*| > 0.7) (Figure 3C). *Anaeroplasma*, *Christensenella*, and *Acholeplasma* showed the most correlations with metabolites. Genera with more than 50% missing values across groups were excluded, leaving 11 genera for further analysis. *Pyramidobacter* was significantly negatively correlated with ADG, whereas *Anseongella* was significantly positively correlated with ADG. *Pyramidobacter* was also significantly negatively correlated with cellulase, cellobiohydrolase, and xylanase activities. *Anaeroplasma* correlated negatively with these enzyme activities, while *Solobacterium* showed significant positive correlations with cellulase and xylanase activities (Figure 3D). Twenty-four metabolites were significantly correlated with ADG (*p* < 0.05, |*r*| > 0.5). Many of these metabolites (e.g., xanthone, rhein, and catechol) were also significantly associated with cellulase, cellobiohydrolase, and xylanase activities (Figure 3E). Correlations between these 24 metabolites and the 11 differential bacterial genera showed that *Pyramidobacter* was negatively correlated with metabolites such as Pipecolic acid, Lignoceric acid, and Sulfaquinoxaline, and significantly positively correlated with Decanoic acid, beta-Hydroxymyristic acid, and Resolvin E1. *Anseongella* was negatively correlated with caffeic acid, ethyl 2-(2-acetyl-3,5-dihydroxyphenyl) acetate, and goniothalenol, and positively correlated with rhein, homopterocarpin, and coumarin (Figure 3F).

## 4 Discussion

Compared with the CON group total mixed ration (TMR), the TRE group exhibited a higher content of acid detergent fiber (ADF), which consists of cellulose, lignin, and silicates. Elevated ADF levels may impair palatability, potentially contributing to the reduced feed intake observed in the TRE group. As feed intake is a key factor influencing animal growth performance (Wang D. D. et al., 2023), its decline could lead to a decrease in daily weight gain.

The stability of the rumen environment directly influences nutrient digestion and absorption. Rumen pH is a key indicator of ruminal stability, with a healthy range typically between 5.9 and 7.2 (Farghaly et al., 2019). In this study, the rumen pH values in both groups remained within the normal range. NH₃-N is produced by microbial breakdown of dietary proteins and non-protein nitrogen compounds; under normal conditions, microbes utilize it to synthesize microbial protein for the host. However, excess ammonia is converted to urea in the liver. Both ammonia nitrogen and microbial protein concentrations were lower in the TRE group. Higher dietary cellulose and lignin in the TRE diet may have impeded protein degradation and reduced ammonia availability (Jin et al., 2023), leading to impaired microbial protein synthesis. VFAs, produced by microbial fermentation of carbohydrates, serve as an energy source for the host and participate in multiple physiological processes such as the tricarboxylic acid cycle, glucose conversion and storage, and lipid synthesis (Ma et al., 2023). Degradation of cellulose and hemicellulose is a major source of VFAs. In this study, lower activities of cellulase, xylanase, and cellobiohydrolase in the TRE group likely contributed to the reduced VFA concentrations, which in turn may have contributed to the lower ADG.

Replacing corn silage with the fermented mixture altered the rumen bacterial community composition, but did not significantly alter its richness, evenness, or dominant populations. Firmicutes and Bacteroidota remained dominant, consistent with previous research (Liu et al., 2023; Jiao et al., 2025; Linde et al., 2023). These phyla play essential roles in ruminal protein and carbohydrate digestion and host energy metabolism (Li J. L. et al., 2025). *Prevotella* was the predominant genus in both groups and is a core rumen taxon in carbohydrate, lipid, and amino acid metabolism (Kou et al., 2024).

LEfSe identified *Anaeroplasma* as the genus with the highest LDA score enriched in the TRE group. As reported by Rey et al. (2023), *Anaeroplasma* may inhibit the fibrolytic activity of certain rumen fungi and bacteria. In the present study, *Anaeroplasma* was negatively correlated with cellulase, hemicellulase, and cellobiohydrolase activities, suggesting its potential involvement in inhibiting fiber degradation, and this inhibitory effect may be associated with the reduction in enzymatic activity. Another study suggested a potential association between *Anaeroplasma* and reduced feed efficiency (Tan et al., 2018). Genera such as *Lachnoclostridium*, *Hungateiclostridium*, *Staphylococcus*, and *Proteiniphilum* are involved in fiber degradation. Specifically, *Lachnoclostridium* breaks down cellulose and related plant cell wall polysaccharides into monosaccharides (Hu et al., 2020); *Hungateiclostridium*, a member of the Firmicutes phylum, degrades cellulose and produces short-chain fatty acids (Jeong et al., 2021); *Staphylococcus*, a major fibrolytic rumen genus, secretes substantial cellulases and hemicellulases, especially xylanases (Wang R. J. et al., 2023); and *Proteiniphilum* enhances the hydrolysis of cellulose and lignin to generate sugars (Sohail et al., 2022). These taxa were significantly enriched in the TRE group, likely responding to higher dietary cellulose and lignin. Despite increased relative abundance of some fibrolytic microorganisms, fibrolytic enzyme activities were lower in the TRE group; this discrepancy may reflect complex microbial interactions and potential inhibition of fibrolytic enzyme activity by taxa such as *Anaeroplasma*. In contrast, genera like *Solobacterium*, *Holdemania*, and *Acetoanaerobium* were more abundant in the CON group and have been implicated in short-chain fatty acids synthesis (Song et al., 2023; Orbe-Orihuela et al., 2022; Böer et al., 2024). The CON group had lower lignin content, which likely enhanced the degradability of its structural carbohydrates and may have promoted short-chain fatty acid synthesis.

Untargeted metabolomics revealed that the TRE diet altered the ruminal metabolite profile, with enrichment in pathways including vitamin B6 metabolism, butanoate metabolism, and biotin metabolism.

Increased 4-pyridoxic acid (a major vitamin B6 catabolite) in the TRE group suggests enhanced vitamin B6 utilization. Vitamin B6 is essential for numerous enzymes (Gholizadeh et al., 2020). Amin and Onodera reported that certain fibrolytic bacteria such as *Ruminococcus flavefaciens* and *Fibrobacter succinogenes* require vitamin B6 for growth (Amin and Onodera, 1998). Thus, enhanced vitamin B6 metabolism in the TRE group might be attributed to higher dietary fiber and proliferation of fibrolytic bacteria. Biotin concentrations decreased in the TRE group. Biotin serves as a coenzyme for enzymes involved in protein, carbohydrate, and fatty acid metabolism, and enhances fiber fermentation (Eom et al., 2024; Abel et al., 2001). Many fibrolytic bacteria depend on biotin (Chen et al., 2011). The reduced biotin concentration may be associated with increased biotin usage by fibrolytic bacteria.

Phenylalanine—an essential amino acid—can be converted into tyrosine under the catalysis of phenylalanine hydroxylase (Jiang et al., 2025; Timperio et al., 2022); it may also be degraded to produce phenylacetate under certain conditions (Lin et al., 2024; Perez et al., 2023). Phenylacetate content increased while tyrosine decreased in the TRE group, and tyrosine metabolism was downregulated with reduced dopa levels. This pattern suggests more phenylalanine was catabolized to phenylacetate rather than converted to tyrosine, which could attenuate downstream pathways such as catecholamine synthesis. Tyrosine can be converted into L-dopa by tyrosine hydroxylase. L-dopa is further decarboxylated by dopa decarboxylase to produce dopamine, which modulates ruminal immune and inflammatory activity by inhibiting NLRP1 inflammasome activation and downstream inflammation via DRD3 signaling (Xu et al., 2024). Moreover, L-dopa serves as a precursor for catecholamines such as norepinephrine, epinephrine, and dopamine, and can stimulate growth hormone secretion (Gao and Geng, 2022). The ruminal concentration of cholic acid, a major secondary bile acid, was significantly lower in the TRE group than in the CON group. Primary bile acids are conjugated with taurine or glycine, stored in the gallbladder, and subsequently converted into secondary bile acids by bacterial action in the intestines (Deng et al., 2024). Bile acids regulate lipid metabolism by promoting the absorption and digestion of dietary lipids, and they are also involved in glucose homeostasis and protein synthesis (Deng et al., 2024; Li M. et al., 2025). The elevated level of 3-hydroxybutyrate in the TRE group further contributed to enhanced lipid metabolism and inhibited glycolysis (Sun et al., 2023). The conversion of butyrate into 3-hydroxybutyrate by mature epithelial cells provides energy to the host and is considered an indicator of rumen maturation and VFA utilization capacity (Deelen et al., 2016).

Within fatty acid anabolic pathways, upregulated metabolites included capric acid and caprylic acid, both medium-chain fatty acids (MCFAs) with chain lengths ranging from 6 to 12 carbon atoms. Previous studies suggest that higher MCFA content can reduce the digestibility of neutral detergent fiber (NDF) (Hollmann et al., 2012). This finding is consistent with our results, in which capric acid was significantly negatively correlated with cellulase, xylanase, and cellobiohydrolase activities.

Microorganisms and metabolites in the rumen not only individually influence host growth and development but also interact with each other (Mao et al., 2016; Yang et al., 2024). Ferulic acid, which increased significantly in the TRE group, is linked to arabinoxylan and lignin via ester and ether bonds, thereby inhibiting cellulose degradation (Zhang et al., 2023). Additionally, ferulic acid monomers can further suppress cellulose degradation by inhibiting microbial growth and the activity of cellulase and other enzymes (Saad et al., 2008; Chesson et al., 1982). Upon release, ferulic acid can be absorbed by the rumen or degraded by microorganisms, thereby mitigating its inhibitory effects (Wang et al., 2022). In this study, ferulic acid concentration was positively correlated with the relative abundance of *Anaeroplasma*. Based on this positive correlation and their shared significant negative associations with fibrolytic enzymes, we hypothesize that *Anaeroplasma* may interfere with the microbial metabolism of ferulic acid, thereby affecting the activity of fibrolytic enzymes. However, this proposed mechanism still requires validation through further studies. Correlation coefficients identified 24 differential metabolites and 2 differential bacterial genera significantly correlated with ADG. *Pyramidobacter* showed a significantly negative correlation with ADG, although previous work has found species within this genus enriched in high-ADG groups (Xu et al., 2024). This discrepancy may stem from two factors: first, different species within the genus *Pyramidobacter* may exert distinct effects on average daily gain, whereas the present study only analyzed its correlation with ADG at the genus level; second, the complexity of interactions within the ruminal microbial community may contribute to this inconsistent observation. *Anseongella* correlated positively with ADG and may be involved in degradation of recalcitrant macromolecules such as cellulose, hemicellulose, and lignin (He et al., 2022). Regarding metabolites, several including homopterocarpin, rhein, resolvin E1, and catechol were significantly correlated with ADG, with the activities of cellulase, xylanase, and cellobiohydrolase, and with the abundances of *Pyramidobacter* and *Anseongella*. This supports the idea that microbe-metabolite interactions modulate relevant enzyme activities, affect nutrient digestion and absorption, and thereby influence host growth performance.

## 5 Conclusion

Contrary to our hypothesis, complete substitution of corn silage with a fermented cotton straw-apple pomace mixture adversely affected the growth performance of beef cattle. Specifically, feeding beef cattle a fermented mixture resulted in significantly lower ADG and DMI, reduced ruminal VFA concentrations, and decreased activities of cellulase, xylanase, and cellobiohydrolase compared with corn silage-fed cattle. This dietary substitution also altered the rumen bacterial community and metabolomic profile. These microbial and metabolic shifts likely suppressed fibrolytic enzyme activities collectively. The resulting limitation energy supply, coupled with reduced DMI, explains the decline in ADG. Consequently, the present findings indicate that a fermented mixture is not yet a suitable complete replacement for corn silage, and further optimization of the fermentation strategy is required to enhance its feeding value.

## Data Availability

The rumen bacteria raw sequencing data have been deposited in the NCBI under accession number PRJNA1332918.

## Footnotes

1
http://www.arb-silva.de
